# Beyond rating scales: With targeted evaluation, large language models are poised for psychological assessment^☆^

**DOI:** 10.1016/j.psychres.2023.115667

**Published:** 2023-12-10

**Authors:** Oscar N.E. Kjell, Katarina Kjell, H. Andrew Schwartz

**Affiliations:** a Psychology Department, Lund University, Sweden; b Computer Science Department, Stony Brook University, United States

**Keywords:** Large language models, Transformers, Artificial intelligence, Psychology, Assessment

## Abstract

In this narrative review, we survey recent empirical evaluations of AI-based language assessments and present a case for the technology of large language models to be poised for changing standardized psychological assessment. Artificial intelligence has been undergoing a purported “paradigm shift” initiated by new machine learning models, large language models (e.g., BERT, LAMMA, and that behind ChatGPT). These models have led to unprecedented accuracy over most computerized language processing tasks, from web searches to automatic machine translation and question answering, while their dialogue-based forms, like ChatGPT have captured the interest of over a million users. The success of the large language model is mostly attributed to its capability to numerically represent words in their context, long a weakness of previous attempts to automate psychological assessment from language. While potential applications for automated therapy are beginning to be studied on the heels of chatGPT’s success, here we present evidence that suggests, with thorough validation of targeted deployment scenarios, that AI’s newest technology can move mental health assessment away from rating scales and to instead use how people naturally communicate, in language.

## 1. Introduction

Recently, artificial intelligence-based (AI-based) language analysis has undergone a “paradigm shift” fundamentally changing how systems are developed in the field (Bommasani et al., 2021). Just a few years ago, natural language systems were primarily purpose-built –statistically optimized for a particular task such that, for example, systems for answering natural language questions (i.e., question answering) used a different model than that for sentiment analysis (scoring the positivity or negativity of a text) or paraphrasing (producing alternative phrases for a small section of text). Now, nearly all AI language systems are built on a *large language model* base or “foundational” model. The state-of-the-art system for sentiment analysis, question answering, paraphrasing, and dozens of other language tasks are based on the same underlying statistical deep learning model, which only needs to be “fine-tuned’’ or adapted to perform particular tasks. In fact, this technology now touches the daily life of nearly everyone with a smartphone, as it has quickly become the basis for modern Web search (Nayak, 2019), digital assistants’ language (Alexa, Siri, etc.), machine translation, and keypad autocompletion. In fact, base models for other domains of AI (vision, speech) are now finding benefits for integrating language models (e.g., Gao et al., 2023; Radford et al., 2023).

The transformer-based Large Language Model is the technology enabling this purported paradigm shift (Bommasani et al., 2021; Devlin et al., 2019). Large language models owe their success largely to their ability to statistically model words in a large context with which they occur by using the *transformer*, a particular deep learning technique (Devlin et al., 2019; Vaswani et al., 2017). Bringing such context to psychological text analysis, large language models can more precisely quantify the specific meaning of language and yield a truer understanding of the person behind the words.

The link between language and psychological phenomena has long been known (Boyd and Schwartz, 2021; Pennebaker et al., 2003; Tausczik and Pennebaker, 2010), and while the use of AI in psychology is not yet widespread, it has been used to successfully gain insights into, e.g., who we are (Argamon et al., 2007; Berger and Packard, 2021; Kwantes et al., 2016; Schwartz et al., 2013), how we feel (De Bruyne et al., 2022; Eichstaedt et al., 2018; Sun et al., 2020), our behaviors (Curtis et al., 2018; Kjell et al., 2021; Macavaney et al., 2021), and other topics (Eichstaedt et al., 2020; Iliev et al., 2015; Jackson et al., 2021; Schwartz and Ungar, 2015). However, quantitative assessment of the primary way humans communicate (language) has yet to reach wide-spread adoption (Boyd and Schwartz, 2021).^1^

**Fig. 1.**
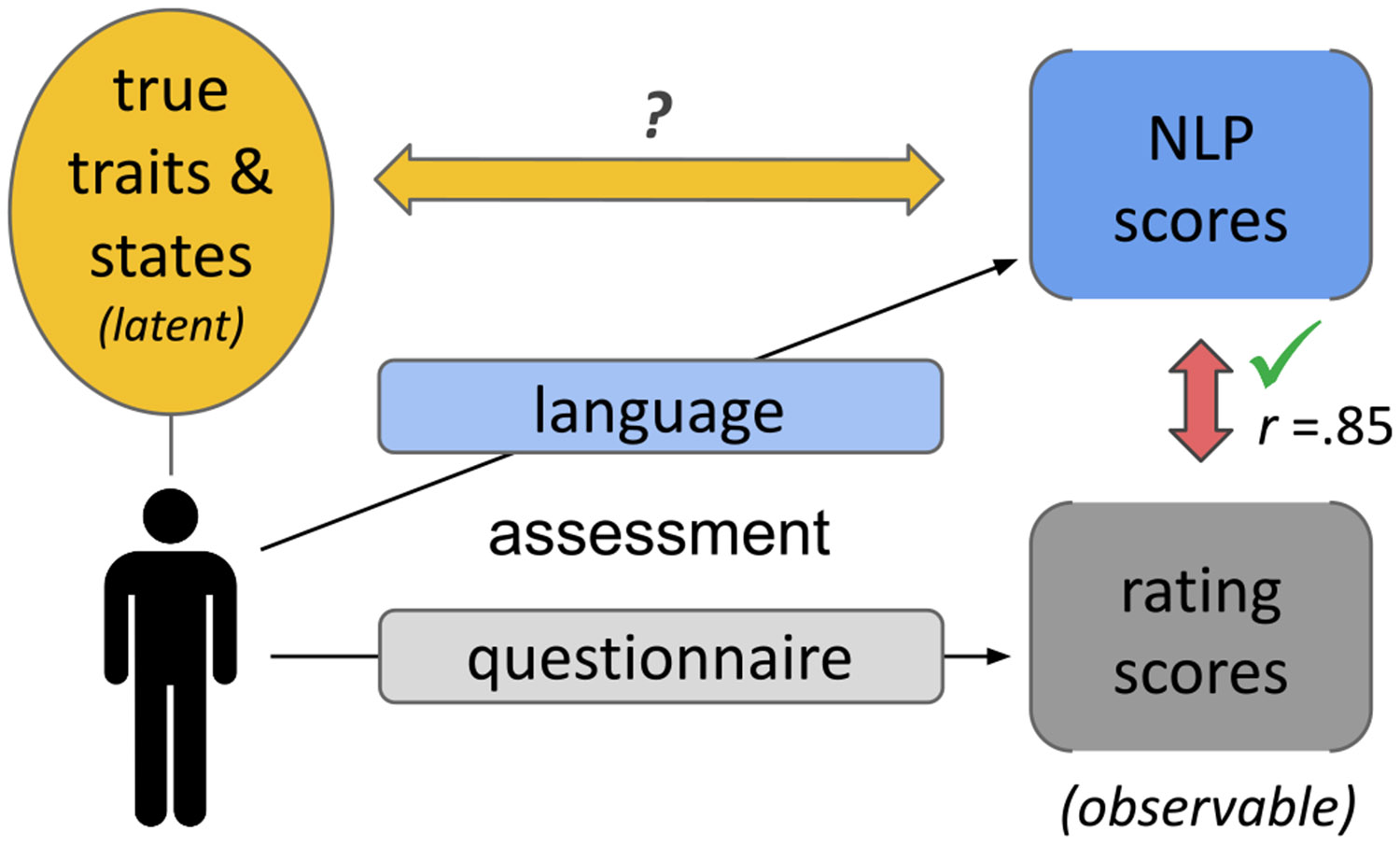
The goal of assessments is to match the **true** psychological traits and states (gold arrow) but most work thus far evaluates against rating scales (red arrow).

Even without large language models, using AI-based language analysis of probed language (i.e., open-ended responses to survey questions), it is possible to derive a quantified score of a psychological construct with moderately high convergence (*r* = *0.72*) with rating scales (Kjell et al., 2019a). *Large language models* push the accuracy to a theoretical upper limit of predicting rating scales, *upwards of r* = *0.85* (Kjell et al., 2022). Hence, early empirical successes in using large language models for mental health assessments (e.g., Kjell et al., 2022; Matero et al., 2019; Mohammadi et al., 2019; Zirikly et al., 2019) suggest that this technique needs not only to change AI (Bommasani et al., 2021) but that it is an essential aspect for an improvement in psychological assessment of mental health (Fig. 1). This suggests that the technique has the potential to modernize assessment methods, from the reliance on closed-ended rating scale responses to *more accurate, fine-grained, and ecologically valid assessments of individuals’ state of mind.* By fully leveraging individuals’ personal descriptions of their mental state in their own words, the technique has the potential to –not only improve current assessments incrementally– but also change the very nature of how individuals’ states of mind are both *measured* and *described* and ultimately increase our understanding of mental health.

Examining the question of whether large language models can modernize psychological assessments on subjective states of mind and experiences beyond rating scales, we are: 1) reviewing the intrinsic advantages of natural language in communicating mental health and showing how language has favorable measurement characteristics, 2) describing how word context matters in mental health, and reviewing how the unique contributions of *large language models* may realize the measurement precision of language, and then (3) provide evidence indicating how these advantages of *large language models* can make the long-held goal of grounding psychological mental health assessment in natural language a reality. Lastly, we discuss biases, risks, and ethical considerations related to using large language models for psychological assessments in research and clinical settings.

Large language models, specifically the application chatGPT, have received much attention in the last year. This narrative review is pre-dominantly not about chatGPT but rather the *AI technology* behind it, which is called *transformers*. The technology of transformer large language models is the focus of the evidence presented in this review for their readiness for psychological assessments. In fact, the transformer language models behind chatGPT (i.e., GPT3.5 and GPT4) are not appropriate for most psychological assessment research because they are closed (not accessible) and often changed (Chen et al., 2023). On the other hand, the majority of transformers we discuss below are open (available for download) and static. Our focus is on reviewing evidence on the application of the technique for *assessment* rather than other psychological tasks, such as in the delivery of automated psychotherapy, which likely needs much more development and has not been empirically validated for safety and efficacy (Stade et al., 2023).

This narrative review focuses on psychological assessment from participant language use (as opposed to wearable or smartphone sensor 7data (e.g., see D’Alfonso, 2020; Melcher et al., 2020; Seppälä et al., 2019). While there is also much interest in large language models in the delivery of interventions or therapy through specialized chatbots (e.g., see Boucher et al., 2021; Parmar et al., 2022; Torous et al., 2021), the technology is much further established empirically for psychological assessment. We further focus on the behavior of natural language use and the subjective experiences language expresses (as opposed to neuropsychological tests; e.g., Chandler et al., 2020) from natural language. The term *language-based assessments,* introduced by Park et al. (2015), refers to the automatic generation of a score for a given psychological construct from observed language use patterns. Importantly, language-based assessments can integrate additional data beyond language (e.g., participant age; Son et al., 2022), and for the scope of this article, we consider it a language-based assessment as long as the language is used in the psychological construct score calculation. Beyond this quantitative assessment, data for language-based assessment lend themselves to additional analyses such as data-driven language-based summaries/depictions of psychological states or traits (e.g., Schwartz et al., 2014; Kjell et al., 2019).

## 2. Intrinsic advantages of natural language

Imagine that a new patient is in the midst of their intake assessments. However, instead of being presented with a slew of questionnaires, they are presented with a prompt for a natural conversation and start talking with AssessmentTransformer (AT), an AI-based social dialog agent.

AT starts: How are you feeling?

Patient: Eh. A bit down. Just having a rest day.

AT: Please elaborate; how are you feeling a bit down?

Patient: I’m tired and not motivated to do anything today. Work is not improving despite raising my concerns. I feel trapped, but I’m putting on a brave face for my family.

An engaging interaction follows, where the patient expresses their psychological traits and states in their natural communication medium. AT has a decent grasp of the psychological meaning of the words being expressed because it understands them in their context –that they have *concerns* with *work*, that *feeling trapped* refers to things not improving, and that *putting on a brave face* does not mean they are literally putting on a mask. After the exchange, AT produces robust quantitative scores for the patients’ mental health (e.g., level of depression, anxiety, stress). In addition, qualitative explanations and descriptions of such assessments provide context to the quantitative scores in the patient’s own words (e.g., the level of depression relates to feeling *down, tired, not motivated,* and *trapped at work*).

Accurate mental health assessments are central to ensuring great care. It is a prerequisite for precision in healthcare: to provide the right treatment for the right person at the right time (i.e., precision mental health; Delgadillo and Lutz, 2020). In addition, accurate assessment is the core for improving care: It is the foundation to systematically develop and secure the quality of care. Intrinsically, natural language as a response format has *ecological validity* –it has long been considered a “window” into psychological states and is our natural way of communicating inner experiences and states of mind (e.g., Tausczik and Pennebaker 2010).

Quantitatively assessing language in an accurate manner has previously been difficult. Likert’s (1932) popular closed-ended rating scale format side-steps this by attempting to capture a *one-dimensional* latent variable of individuals’ attitudes (which has been generalized to assess psychiatric disorders and experiences more broadly). However, besides this being an unnatural way for people to communicate their states of mind, the scale reduces possible responses to a relatively narrow, fixed range and a constrained resolution –there is only a finite number of possible responses and scores. With sufficient development cycles, advances from Classical Test Theory and Item Response Theory have introduced methods to better select items and aggregate closed-ended responses into latent variables thought to represent true states and traits better (Lord, 2012; Thomas, 2011). However, the closed-ended nature necessitated by these methods does not allow respondents any flexibility in expressing a state of mind that deviates from the posed items –complex or unusual views are lost. They are also still limited to the inherent information loss of the item-response format. Compared to open-ended natural language, the rating scale method is overly reductionist.

### 2.1. Information-rich language

The idea of capturing more information can be formalized via information theory (Shannon, 1948), whereby a mathematical concept of *self-information* can be measured as the amount of *diversity* that can be represented in a dataset. Self-information is a key measure in machine learning as it shows the amount of information that algorithms have at their disposal to learn. The greater the self-information, in general, the greater the expected ability to predict variables from a given dataset (MacKay, 2023). For example, a *yes*/*no* item that is answered 50 % *yes* and 50 % *no* in a dataset will have more *information* than one that is answered 90 % *yes* (because the latter does not distinguish patients as well). Data from more item responses yield more *information* only if answered in a way that cannot be mapped from the other items. Similarly, more words in natural language responses do not automatically yield more *information*; in fact, it turns out that individuals’ description of their harmony in life using ten descriptive words (mean = 9.8 words/response) yield more information than their corresponding text responses (mean = 69 words/response; Kjell et al., 2022). However, to our knowledge, a comparison between natural language and rating scale responses has not been done.

To examine the difference in *self-information* of natural language versus rating scale responses assessing general affect, we asked 100 participants^2^: *How are you feeling?* Leaving an open response box. This was followed by the commonly used closed-ended rating scale, the Positive and Negative Affect Schedule (PANAS), asking respondents to describe their “feelings and emotions” using 20 affect-related items rated from 1 = “very slightly or not at all” to 5 = “extremely”.

**Fig. 2.**
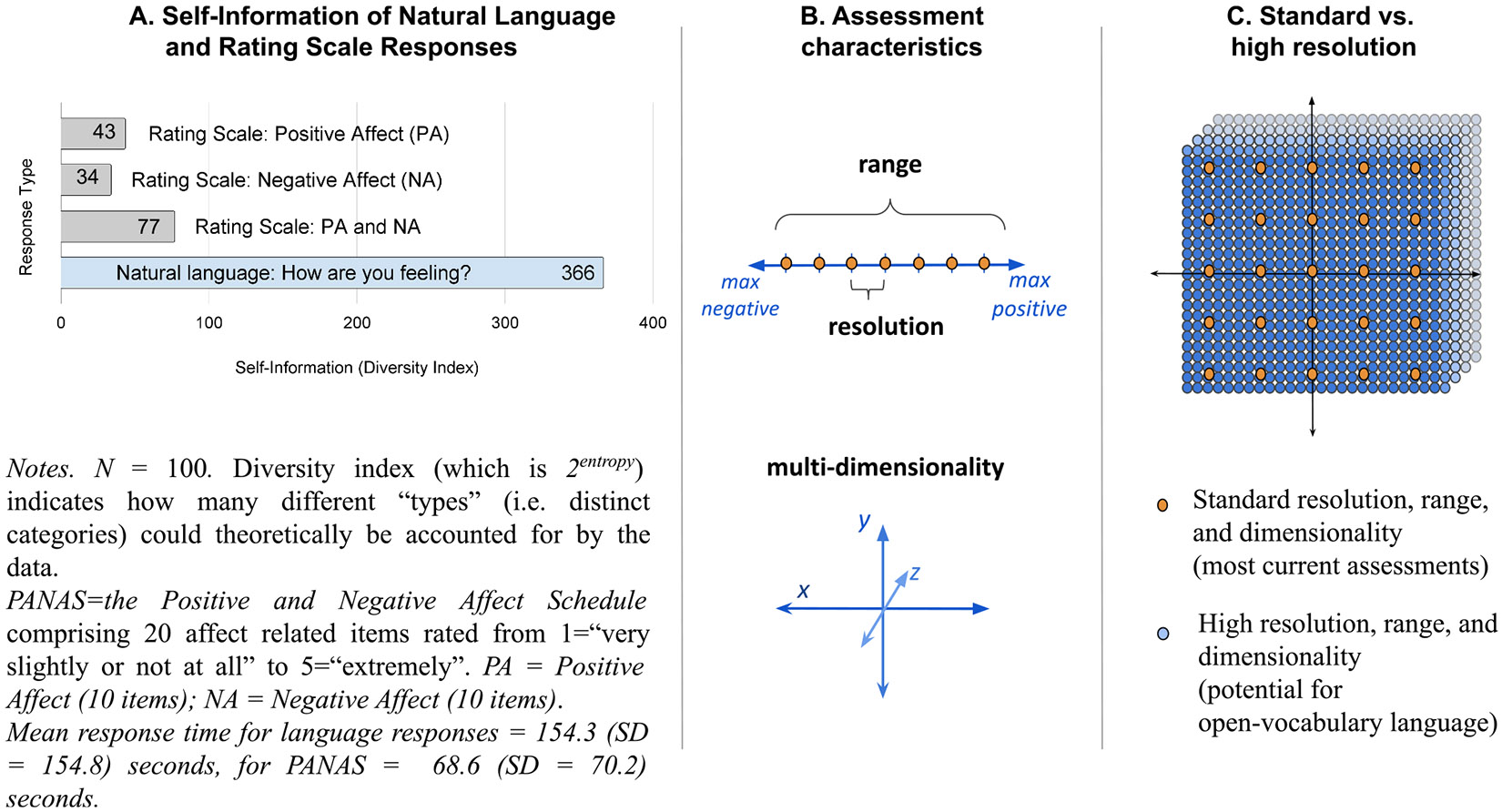
**A. Comparison of self-information between response formats for affect.** Self-information (also known as the Diversity Index) indicates a theoretical minimum number of bits necessary to represent the data. Natural language responses contain more than four times as much information than rating scales. **B. Depiction of characteristics of assessment response data that can lead to greater information content.** The assessment characteristics include *range* (i.e., the lower and upper limits), *resolution* (i.e., the smallest measurable interval) and *multi-dimensionality* (i.e., including several dimensions). Language has the ability to be greater in all three characteristics. **C. Illustration of standard versus high *resolution, range* and *dimensionality* measurement in coordinate space.** The standard resolution demonstrates 2 dimensions with a range of 5 values, while the high resolution demonstrates a 3rd dimension (in reality, it may have many more) with greater range and resolution.

Applying a self-information measure (the Diversity index^3^) to both response formats (Fig. 2A) demonstrates 4.8 times more self-information from the natural language responses (Diversity Index = 366) as compared with the rating scale responses (Diversity Index = 77). Thus, the natural language response tells us more, yielding a greater ability to distinguish responses than the full PANAS item-response scale. As such, language comprises many favorable measurement characteristics, including high *range, resolution, dimensionality* (Fig. 2B, C), and *openness*.

The *range* of language enables us to describe the extremes (absolutely loves and hates), while its *resolution* yields nuanced differences between (cherishes, loves, adores, likes). The *multi-dimensionality* of language affords efficient and detailed communication of complex states of mind (love, excitement, joy, awe), which are not constrained to one dimension. The *openness* of natural language also enables us to creatively and fittingly construct personal ways of describing our state of mind (e.g., choosing *adoration* or *despise* rather than *love* or *hate*, or communicating multi-faceted descriptions for a situation that researchers or clinicians may not have anticipated: *it was rough but it’s over now*).

Of course, that natural language responses comprise more information than rating scales, does not necessarily mean they yield better measurement of any particular construct –the information may not be relevant to the psychological construct. Next, we focus on how large language models also deliver on psychological construct-relevant information.

## 3. Context matters

An important aspect of language is that words take on different meanings depending on context. Understanding words in their context computationally (word sense disambiguation) has been considered “an AI-complete problem, that is, a task whose solution is at least as hard as the most difficult problems in artificial intelligence” (Navigli, 2009). The ability to contextualize word sense is essential for capturing different psychological dimensions. For example, consider the italics words in the responses from Person A and B:

**Table:** 

	Person A	Person B
*How are you?*	I feel *fine* –even *great*!	My life is a *great* mess! I’m having a very hard time being happy.
*What is going on?*	Earlier, I *played* the *game* Yahtzee with my *partner*. I could not get that *die* to roll a 1! Now I’m *lying* on my bed for a *rest*.	My business *partner* was *lying* to me. He was trying to *gam*e the system and *played* me. I think I am going to *die* –he left and now I have to pay the *rest* of his *fine.*

The meaning of *play* differs in the examples, from *amusing recreational activity* (Person A) to *being taken advantage of* (Person B). One would not be hard-pressed to be convinced that each has a different psychological meaning –the affective valence ranges from likely positive (Person A) to likely negative (Person B). In fact, according to the popular dictionary WordNet (Miller, 1995), *play* has at least 52 senses. When analyzing words outside of context, they are strikingly ambiguous, which is especially true for frequently used words that tend to have considerably more senses (Resnik, 1995).^4^

The different word senses brought out from the contexts have important connotations for the meaning and thus also for psychological insights. The word *order* within a context is also important; for example, consider the difference in meaning between ”the patient loves the therapy session with the therapist” versus “the therapist loves the patient in the therapy session”; as ChatGPT notes: “In the first, … the focus is on the patient’s feelings and their positive experience of the therapy session”, while “In the second statement, … the focus is on the therapist’s feelings and their positive connection with the patient” with the additional context “it is not appropriate for a therapist to have romantic or sexual feelings for their patient”. While the goal of integrating context into language for psychological analysis has been sought previously (Landauer, 1999; Schwartz et al., 2013; Tausczik and Pennebaker, 2010), it was not possible to achieve effectively for every mention of a word prior to the large language model.

### 3.1. The development of contextual word representations

**Fig. 3.**
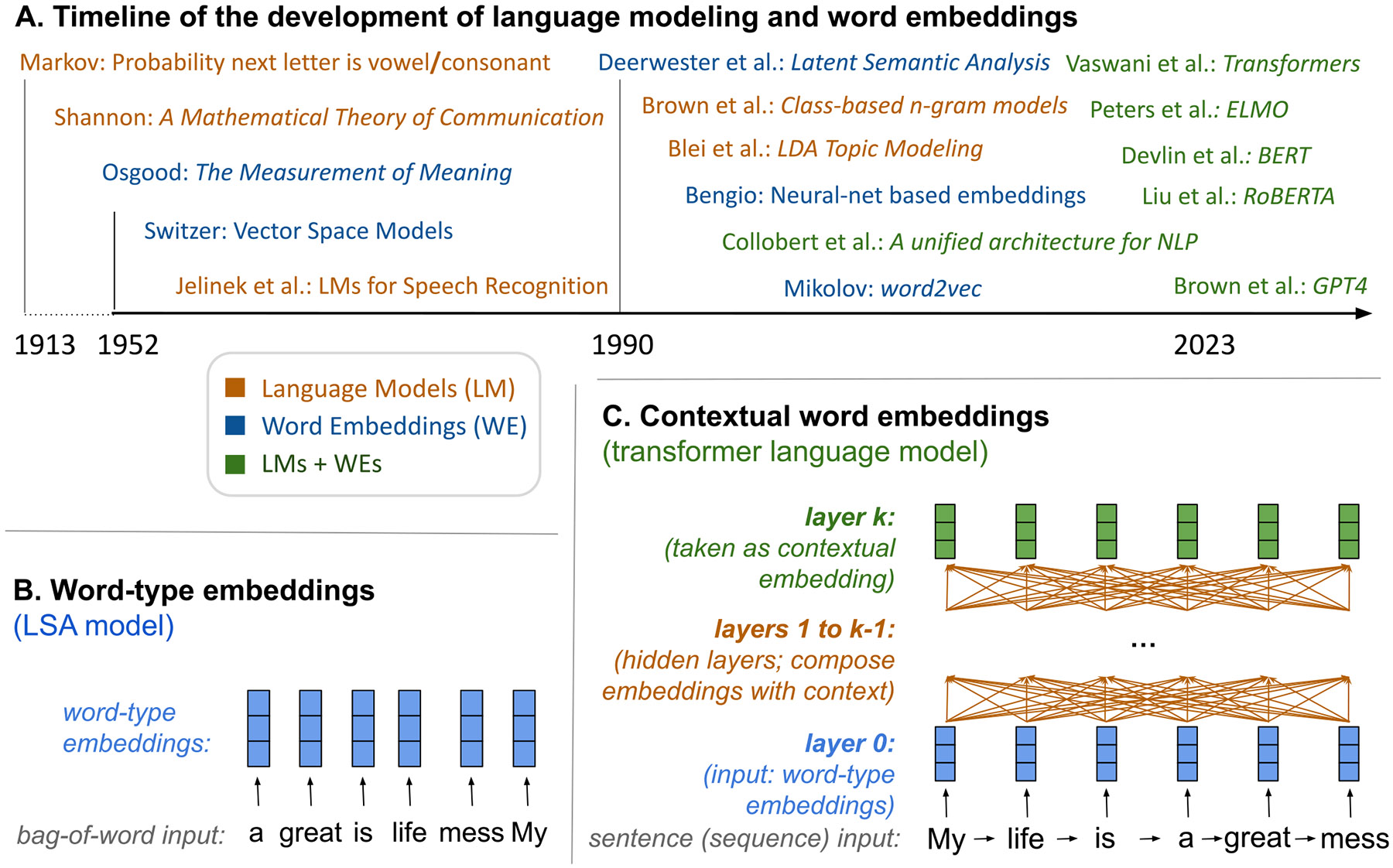
**A. The timeline of the development of transformer-based language models.** Transformer-based language models came out of work on language modeling (i.e. the task of estimating the probability of word in their context) and vector semantics (i.e. an approach to representing the meaning of words or phrases as an array of continuously valued numbers). Transformer language models represent words in their context as continuously-valued numbers optimized for the task of language modeling. **B. Depiction of embedding the sentence “My life is a great mess” using traditional word type embeddings.** Word-type embeddings always represent the same word with the same vector. In this sense, when they are applied, they have no notion of context. **C. Depiction of a contextual word embeddings from transformers of the same sentence.** Such embeddings start with static representations from word-type embeddings but then proceed to produce new embeddings that consider the other words in context. For example, the representation of “great” not only depends on itself but what comes before and after, such as “mess”. Contextual word embeddings utilize a different representation for each instance of a word depending on their context and therefor they can encode the more precise semantics or meaning necessary for language modeling. Note: Refs. “Bengio et al. (2003), Blei et al. (2003), Brown et al. (1992), Collobert and Weston (2008), Jelinek et al. (1975), Markov (1913), Osgood (1952), Mikolov et al. (2013), Switzer (1964).”

Word embeddings are needed to turn language data (i.e., lists of letters) into a quantitative form that captures word meaning with which statistical techniques can directly be applied. The fundamental approach to word representations is to map each word to a list of numbers (i.e., *word vectors*). The idea of numerically capturing words’ meaning took off in the 1950s with the convergence of ideas from psychology, linguistics, and computer science (Fig. 3A; Jurafsky and Martin, 2020). Until recently, most methods utilized a *bag-of-words* approach whereby the order of the words in context is not taken into account (an obvious oversimplification that is nevertheless often useful). This method can be contrasted with ordered context from positional embeddings, which enable encoding implicit syntactic structure. *Large language models* are the product of a long-term goal within AI to go beyond “bag-of-words” (e.g., see the predecessors to BERT called ELMo, Peters et al., 2018).

Large language models’ success in going beyond bag-of-words approaches is attributed to deep neural architectures/algorithms, advances in specialized hardware, large language datasets, and their algorithms enabling large training sizes.^5^ The technique enables capturing non-linear relationships of how words relate and interact with each other. An important part of the training process of *large language models* involves predicting a missing (masked) word within a given sequence of words. To succeed with the prediction, the model needs to learn syntax and associations among words; so the model learns from how the words are used in the training dataset. In addition, the *large language models* algorithm relies largely on *attention*, a mechanism that weights the effect of context words on a target word in a given sequence. Hence, attention enables the representation of the relationship between words in a sequence, which can capture long-term information, dependencies, and interactions of words in a text (Fig. 3B, C).

### 3.2. Large language models

The first widely adopted large language model is called BERT (short for Bidirectional Encoder Representations from Transformers; Devlin et al., 2019). Released for open use by Google in 2018, BERT has been followed by a family of large language models, including RoBERTa (Liu et al., 2019), GPT3 (Brown et al., 2020), and XLNet (Yang et al., 2019).

*Large language models* brought about unprecedented accuracy increases across a wide range of standardized Natural Language Processing tasks (NLP; AI’s subfield on language analysis), even surpassing the non-expert human baseline. Language models are typically evaluated and compared on a variety of different tasks, where two of the most common collections of standardized tests and benchmarks include: The *General Language Understanding Evaluation* (GLUE; Wang et al., 2018) and the *SuperGLUE* (Wang et al., 2019).

The GLUE suite comprises nine, and the SuperGLUE eight, carefully selected, standardized language understanding tasks.^6^ The tests include diverse tasks such as sentiment prediction, paraphrasing, similarity, grammar control, word sense disambiguation, causal reasoning, common sense reasoning, reading comprehension, natural language inference, and question answering. With the diverse set of tasks, GLUE and SuperGLUE favor language models that demonstrate ”general-purpose language understanding” (Wang et al., 2019).

At this moment (September 2022), there are 20 different models that surpass the performances of humans. Table 1 presents examples of standardized NLP tasks from GLUE along with person-level language tasks (such as assessing depression and suicide risk as presented later), describing top-performing approaches and their performance. All top-performing approaches include large language models for both standard NLP tasks and person-level tasks.

### 3.3. Leveraging big data information for small samples

The typical focus in NLP is to model language itself using huge amounts of data and employ these language models to solve language tasks (e.g., GLUE tasks). However, *human-level AI* models the individual behind the language (Ganesan et al., 2021; Soni et al., 2022). Modeling a person behind a text may include assessing their depression or suicide risk (Ganesan et al., 2021). Importantly, a huge amount of participant-generated data is *not* required to apply large language models in clinical sciences. First, small language samples can be submitted to a pre-trained model based on a large language model, trained for producing a psychological score. Existing large language models can be downloaded and applied using both Python (e.g., see DLATK; Schwartz et al., 2017) and R (e.g., see the *text-package*; Kjell et al., 2023). Second, developing predictive models with relatively small sample sizes can be achieved through dimensionality reduction of word embeddings (Ganesan et al., 2021); this has, for example, been done when predicting demographics (age, gender), personality (extraversion, openness), and mental health (suicide risk), with as low as 50 participants (and results approaching large-scale model accuracies with as few as 500 participants).

Further, large language models can handle multiple languages, such as multilingual BERT (mBERT), which was trained on the top 104 languages on Wikipedia, from English and Mandarin Chinese to Aragonese and Tagalog. For mental health researchers and practitioners, this not only opens up the possibility to use the techniques in many different languages but also the potential for new types of cross-cultural research; for example, mBERT has been employed to study misinformation about COVID-19 and health on social media in English, Arabic, and Bulgarian (Panda and Levitan, 2021).

Pre-trained language models can also be “fine-tuned” by continuing to train them on domain-specific language. For example, clinicalBERT (Alsentzer et al., 2019) is based on BERT and bioBERT (trained on biomedical text; Lee et al., 2020) with additional fine-tuning on clinical health text data such as notes from clinicians and discharge summaries. As a result, clinicalBERT provides word embeddings that perform more accurately in several tasks related to mental health.

## 4. Psychological insights through contextualized language

Contextualized language is the most common way of expressing and understanding complex psychological phenomena. Language plays a central role in processing and structuring emotions and thoughts introspectively and in communicating them to others. Language helps us sort memories to remember the past and plan the future. We cooperate, learn, and teach through language. Language can also be destructive and violent: A tool in arguments, manipulation, and deceit. These are all central parts of human life –so ignoring the context and structure of language data misses vital information and reduces ecological validity.

### 4.1. Ecological contextualized language

*Large language models* have been instrumental in recent AI models predicting clinically relevant outcomes from individuals’ naturally occurring text. This is demonstrated in the recently *shared tasks* of predicting suicide risks (*N* = 621) from *Proceedings of Computational Linguistics and Clinical Psychology* (CLPsych). The computer science research community has a tradition of arranging shared tasks, where researchers work on common tasks with the same datasets to develop competing methods to identify mental health disorders and related issues: The winning model(s) of a shared task is typically considered state-of-the-art. Prior to *large language models,* deep learning techniques were not able to benefit language-based predictions of mental health tasks (Lynn et al., 2018). In contrast, the two top-performing models included *contextual word embeddings* in the CLPsych shared task in 2019 (Matero et al., 2019; Mohammadi et al., 2019; Zirikly et al., 2019). The shared task included 15 research teams and focused on the extremely difficult task of predicting individual suicide risk from (de-identified) Reddit data, where large language model-based techniques were able to reduce error by 12.7–56.6 % over strong baselines.

The modeling of emotional processes in psychotherapies has also been improved by using large language models to assess the dynamics of valence in transcripts (human ratings from a database of 97,497 utterances). The large language model BERT was trained to predict the mean valence of transcripts rated by experts. The model’s inter-rater reliability with the rated mean (kappa = 0.48) surpasses previous state-of-the-art sentiment models (kappa = 0.31), LIWC (kappa = 0.25) and even the *average* human performance (kappa = 42; Tanana et al., 2021). This is further evidence of a shift where the latest in AI techniques (i.e., deep learning), in general, started giving a win in NLP for psychology.

### 4.2. Probed contextualized language

It might not come as a surprise, but none of the participants in our study communicated their response to the question: *How are you feeling?* with a numeric response (e.g., *I’m a 7 on a scale from 1 to 10*). Neither did anyone only use descriptive words meant to be interpreted without any context (e.g., a list of words: *happy, excited, balanced*). All respondents used natural language to describe their state of mind, and in fact, only 13 of the 100 participants used at least one of the words from the items of the PANAS rating scale commonly used to measure feelings. The main focus of *self-report assessments* is to *measure*/*quantify* the degree of a psychological construct. The typical rating scales of self-report measures comprising frequency labels (e.g., *0*=*Not at all* to *4*=*Nearly every day*; Kroenke and Spitzer, 2002) or agreement labels (e.g., *Strongly disagree* to *Strongly agree*) can be replaced by probes for language. Kjell et al. (2019a) found that probed language-based assessments of well-being and mental health can produce scores correlating with closed-ended rating scales upwards of Pearson *r* = 0.72 (*N* = 477).

Using *large language models,* language-based assessments have been shown to approach the theoretical upper limits in convergence with standard psychological assessments of well-being –the measures’ own *reliability*. Kjell et al. (2022) achieved a Pearson *r* = *0.85* (*N* = 608) for language-based assessment with corresponding rating scale scores for the harmony in life scale. This correlation is stronger than the scales’ own inter-item-correlation average (*r* = 0.76), test-retest reliability (*r* = 0.71–.77), and it is in line with the item-total average correlation (*r* = 0.84). Using *large language models* particularly improved the prediction from text responses rather than descriptive word responses: AI language analyses are now at the point where a quantitative score can be derived from natural language responses without sacrificing accuracy as measured by rating scales. The large language models behind this advance can become a widespread alternative in digital mental health, an avenue for modernizing the self-report of mental health assessments and ultimately improving our understanding of psychiatric conditions and human experiences more broadly.

Contextualized word embeddings have also achieved higher accuracy than trained clinicians and non-contextualized embeddings in classifying individuals diagnosed with schizophrenia (*n* = 30) and healthy individuals (*n* = 30; Sarzynska-Wawer et al., 2021). All three methods assessed transcribed interview answers in Polish to six questions about the participants’ lives and thoughts. The contextualized word embeddings (based on ELMo) achieved an accuracy of 80 % in distinguishing patients from healthy individuals, whereas clinicians assessing the same text only obtained 74 % accuracy. The model based on non-contextualized embeddings only achieved an accuracy of 70 %, which was significantly lower than the contextualized embeddings in a post hoc pairwise comparison (*p* = .03).

### 4.3. Beyond rating scales

We have discussed how natural language possesses high ecological validity (is the natural way of communicating complex psychological constructs), is information-rich (provides more information than rating scales), and comprises many favorable measurement characteristics (high range, resolution, dimensionality, and openness). Next, we describe three additional aspects central to how language-based assessments have the potential to move beyond rating scales, including i) being validated beyond convergence to rating scales, ii) providing descriptions to contextualize scores, and iii) better understanding response contexts (broadened to encompass *who* says *what* to *whom, where*, and *how*).

#### 4.3.1. Beyond rating scales: true scores

Language responses contain valuable information that *large language models* can extract into scores converging with validated rating scales (Kjell et al., 2022). However, rating scales themselves are only an observable “proxy” and not a perfect true score (Fig. 1). Most psychometric theories, such as classical test theory (e.g., Novick, 1966) or item-response theory (Reise and Waller, 2009), view self-report responses as an approximation of the *true* latent variable that is sought. Therefore, the validity of language-based assessments and rating scales should go beyond evaluating their convergence.

This has so far only been studied in a few studies; one such study compared the two methods’ ability to accurately categorize external stimuli of pictures depicting facial expressions including sad, happy, and contemptuous (Kjell et al., 2019a). It was found that language-based assessment (based on the bag-of-words approach) more accurately categorizes facial expressions than rating scales. Further, a study focusing on theoretically relevant behaviors –cooperation– to harmony in life showed that language-based assessments significantly correlated (Pearson’s *r* = 0.18, *N* = 181; and *r* = 0.35 in individuals categorized as prosocials) with cooperative behaviors, whereas the corresponding harmony in life rating scale (Kjell et al., 2016) did not (Kjell et al., 2021b).

The intrinsic advantages of natural language and its favorable measurement characteristics are suitable for data-driven insights – however, the assessments need to be accurately grounded (i.e., models need to be trained to accurate assessments). Accurate assessments of psychiatric symptoms and diagnoses are important but often difficult to achieve. A method to improve assessments when there is no single, error-free measure is to involve experts that assess multiple types of (longitudinal) data to attain increased assessment accuracy (i.e., a best-estimate assessment; Eijsbroek et al., 2023; Leckman et al., 1982; Spitzer, 1983). The potential of this method for training natural language to best-estimate assessments led us to develop a reporting guideline with the aim of helping researchers plan, report, and evaluate such studies (Eijsbroek et al., 2023).

Natural language responses can also be used directly for precision in healthcare: To predict the likelihood of intervention success for a person in time without first predicting a psychiatric condition (e.g., DeRubeis et al., 2014). It can also be used for data-driven insights related to biological markers such as cortisol or relevant behaviors such as sleep patterns.

#### 4.3.2. Beyond rating scales: descriptions more than a score

**Fig. 4.**
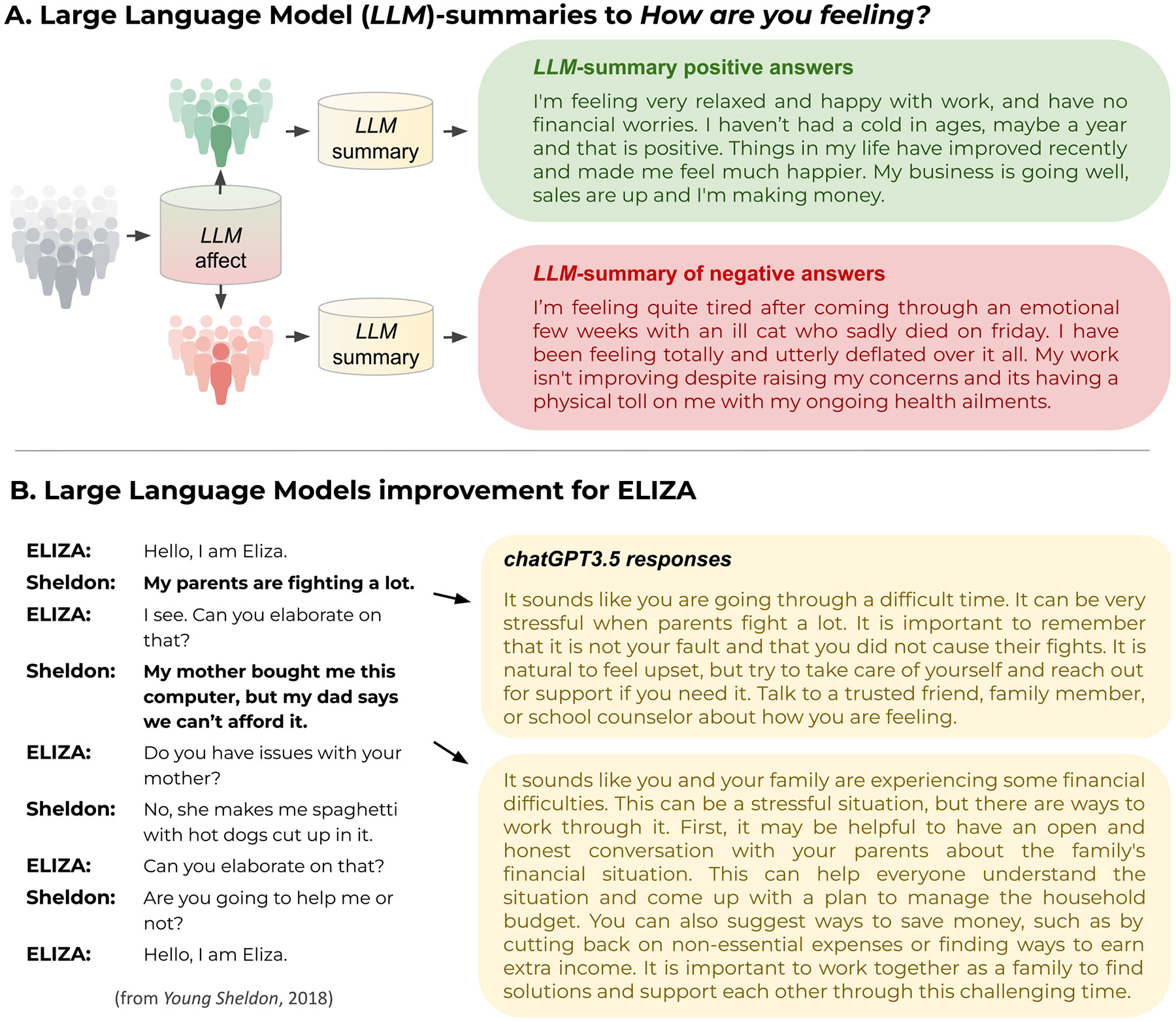
Beyond Rating Scales **A. Large Language Models summary of language associated with positive and negative affect scores.** This is a demonstration of how Large Language Models can be used for differential language analysis. We see that the summaries include both broad statements (“feeling very relaxed”) as well as specific examples of life events (an ill cat passing away). To produce these, affective valence of each response to *how are you feeling?* was estimated using an AI valence estimator and then groups of those classified as positive and negative were fed separately to the large language model *t5-large*^7^ to be summarized. **B. Depiction of output from an early chat system versus a modern Large Language Models based chat system** (ChatGPT3.5, from January, 2023). The left column shows an interaction between Sheldon and the computer software ELIZA from the TV series Young Sheldon^8^. The right column shows responses to the same first two questions by chatGPT3.5. ELIZA, designed by Weizenbaum (1966), is a real computer program that, despite not processing language beyond looking for simple patterns, is quite effective at evoking responses. This demonstrates the ability of large language models to interact with psychologically relevant output.

Language-based assessments also have the ability to be self-descriptive, moving beyond mere scores as the output. For example, statistically significant descriptive words and key phrases can be visualized based on their underlying meaning along relevant dimensions such as low versus high scores of personality traits (Schwartz et al., 2013), depression (K. Kjell, Johnsson, et al., 2021), or in relation to behaviors such as cooperation (Kjell et al., 2021). Fig. 4A shows AI-generated summaries of the ten most negative and positive answers to *How are you feeling?* from our study.^7^ Fig. 4B demonstrates large language models’ power to understand contextualized language and produce psychologically nuanced content.

#### 4.3.3. Beyond rating scales: the expansion of contexts

The meaning (of words) depends on contexts: We have provided examples of how the surrounding words in a text define the meaning of words. Nevertheless, language is contextual beyond itself: “Language arises in the life of the individual through an ongoing exchange of meanings with significant others” (Halliday, 1978). Whereas rating scales offer a restricted response format, consisting of closed-ended options that limit the range of expression, natural language can provide broader contextual information. These contexts can, for example, be broadened to encompass *who* says what to *whom, where,* and *how*.

The *who* may involve psychological contexts and demographic variables. Extraverts, for example, use language differently from introverts (Schwartz et al., 2013). The individual level has been modeled by fine-tuning large language models to keep track of what a specific individual has written previously, which results in word embeddings that more accurately predict human-level variables (Soni et al., 2022).

The *whom* may involve social contexts such as personal, professional, or healthcare settings that prompt individuals to describe and express themselves differently. Note also that response interface contexts, such as a questionnaire, virtual (e.g., a chatbot), or physical (e.g., a robot) interfaces may influence responses. The *where* may involve situational contexts such as how being in a waiting room, on a bus, or at home may spark different answers. Research comparing individuals’ willingness to disclose in health-screening interviews found that individuals led to believe a Virtual Human was computer-controlled, as compared to human-controlled by an operator, showed a higher willingness to disclose (Gratch et al., 2014). The participants in the computer-controlled context also displayed more intense feelings of sadness and lower impression management.

The *how* may consider the language-eliciting context, such as passive or prompted language, and the medium including written or spoken language. In addition, physical contexts, including facial expressions and body language, may significantly interact with the meaning of words.

Many of these contexts are potentially important to consider when collecting and analyzing data – and the generalizability of specific models to other contexts. More research is needed on best practices and the optimal way of taking advantage of contextual factors in assessing psychiatric conditions.

## 5. Targeted validation for deployment scenarios

While lots of evidence now exists that the technology is capable of strong validity (convergent, discriminant, external criteria; Kjell et al., 2021b, 2019, 2022; Oltmanns et al., 2021; Son et al., 2021), there are currently no “one-size fits all” models that have been validated across multiple populations or conditions, and only a few models have been tested for particular clinical deployment situations (Eichstaedt et al., 2018; Kelly et al., 2022; Son et al., 2021). Deployments of language model software for specific clinical use cases require supporting evidence for validity and reliability (as is needed for rating scales too). Hence, it is important to distinguish that this narrative review provides support for large language models for assessment as a class of techniques or algorithms. However, it does not advocate that all instances of such models should be trusted for assessment.

As a class of techniques, large language model assessments have demonstrated validity and reliability on par or better than rating scales, but any particular instance of such an assessment should go through a thorough evaluation for validity (including bias) and reliability before use in research or clinical practice just as any rating scale should. This involves validating models for *target populations* and *use-contexts* (for example, see studies analyzing language from clinical settings such as therapy sessions (Tanana et al., 2021), general online surveys (Kjell et al., 2022), social media such as Facebook (Lynn et al., 2020), Reddit (Zirikly et al., 2019), and Twitter (Coppersmith et al., 2015) as well as *use-cases* (such as screening (Sawhney et al., 2020) or diagnostics (Eichstaedt et al., 2018)). Assessment models also need to be validated beyond cross-sectional contexts, where studies to date have analyzed language use over time for assessment (Eichstaedt et al., 2018), capture dynamic changes (Schwartz et al., 2014; Tsakalidis et al., 2022) and predicting future symptoms trajectories (Son et al., 2021).

It is also important to control for demographic variables to understand the validity of language-based assessments beyond variables such as age, gender, and socio-economic status. It has, for example, been demonstrated that Facebook language (AUC = 0.69) predicts depression in medical records more accurately than demographic characteristics (age, sex, and race, AUC = 0.57, Eichstaedt et al., 2018). Further, studies have demonstrated that language-based assessments provide improved accuracy when controlling for occupation, age, and gender (Son et al., 2021), seven socio-economic variables such as household income and education (Matero et al., 2023), and gender and social class (Lynn et al., 2018).

## 6. Biases, risks, and ethical considerations

The weaknesses and strengths of *large language models* come with ethical considerations and responsibilities. Since this research is often interdisciplinary and involves complex methods, it is essential to develop rigorous frameworks guiding scientific evaluations of such studies (Chandler et al., 2020). Chandler et al. (2020) provide a framework for addressing common issues in integrating and evaluating the potential use of AI in psychiatry; the framework emphasizes i) the importance of evaluating the explainability of models, ii) assessing the transparency of the method, and iii) ensuring that the AI model generalizes. These issues apply to language-based assessments; it is, for example, important to explain how the model weights different parts of a text, be transparent in model architecture, training data, and performance, as well as ensure generalisability by using large representative datasets to test models in.

The section on using large language models for psychological assessments references results based on *open* large language models, meaning that a model comes with detailed documentation, can be downloaded, shared with others, and run offline (e.g., BERT, RoBERTA, and BLOOM). So providing sufficient information about the models, including the exact version, where it can be downloaded, the number of layers that were used, and how, will enable others to reproduce the results (which, of course, is facilitated if combined with open code). Openness, however, is not always the case with recent state-of-the-art *closed* models. ChatGPT, for example, cannot be downloaded and used offline, and it is only partly documented: The exact architecture and training data is not revealed, and independent investigations suggest that the model is often updated without the possibility of accessing previous models (or the exact differences; Chen et al., 2023). The closedness comes with scientific and ethical concerns. Closed models result in issues relating to replicability (i.e., a model version update can even prevent researchers in charge of the original analyses from replicating their results). From an ethical perspective, when a model cannot be downloaded and run offline, they require the user to share their data with the model host. Hence, researchers must be very cautious in sharing sensitive data with companies hosting a closed model.

Leidner and Plachouras (2017) specifically discuss ethical challenges to NLP, where they emphasize that ethical values should be incorporated in both the development and the application of NLP; this, for example, includes considering biases in large-scale language models (Kurita et al., 2019; Shah et al., 2020), privacy in regard to the open-ended language format and increased predictive power. Thus, it is important to undertake extra privacy-preservation steps, including extra data access restrictions and thorough checks for identity if seeking to share data (see Lison et al., 2021, for a thorough discussion).

There is a growing interest in ethical principles and frameworks for developing and deploying AI (Jobin et al., 2019; Peters et al., 2020), with an active debate about best practices. An extensive review of over 80 international guidelines on ethical AI, revealed five global ethical principles, including *transparency, justice and fairness, non-maleficence, responsibility*, and *privacy;* however, there are currently substantial differences in how they were conceptualized and how they should be applied (Jobin et al., 2019).

There are also legal and regulatory frameworks to consider when developing and implementing AI techniques in clinical settings. The European Commission (2023) typically requires AI solutions for clinical settings to be CE-marked (a certification declaring high safety and health requirements); they have also proposed regulations specifically concerning artificial intelligence –the AI Act– seeking to harmonize rules for the development and use of safe AI (see also Veale and Zuiderveen Borgesius 2021, Hauglid and Mahler 2023). In the United States, the Food and Drug Administration (FDA, US Food and Drug Administration, 2021) provides regulations for the application of AI in clinical settings, and the White House Office of Science and Technology Policy (White House Office of Science and Technology Policy, 2022) released a blueprint for an AI bill. This blueprint proposes five principles to guide the use of AI, including i) safe and effective systems, ii) algorithmic discrimination protections, iii) data privacy, iv) notice and explanation (i.e., informing consumers when and how AI is being used), and v) option to opt-out, with human alternatives, consideration, and fallback. These regulations and guides are quickly being updated, and it is out of the scope of this article to describe these resources at length.

## 7. Conclusions

This narrative review suggests that the more precise language scores provided by *large language models* can change how mental health is assessed by enabling patients and study participants to respond in their own words, resulting in large improvements in accuracy as well as an expanded scope of insights. Many studies already collect open-ended responses for qualitative review, and such techniques can be used to complement traditional rating scales while they are being established. Further, many resources are assisting in enabling the application of these methods to mainstream mental health research (Python library DLATK; Schwartz et al., 2017; R-package *text*; Kjell et al., 2021). This body of work suggests that AI’s paradigm shift to *large language models* (Bommasani et al., 2021) can lend itself to a change in psychology from the mostly ubiquitous reliance on rating scale responses to a more accurate, fine-grained, and ecologically grounded assessment from fully leveraging participants’ own words.

## Figures and Tables

**Table 1:** Examples of Standard NLP and Person-Level Language Tasks: Top Performing Systems and their Performance.

Standard Document-Level NLP tasks (GLUE)			Person-Level Psychological Tasks		
Task	Top performing approach^A^	Performance	Task	Top performing approach	Performance
Sentiment (SST-2) *Is a review of a movie positive or negative?*	Large language models-architecture for large-scale knowledge enhanced pretraining (EARNIE^1^)	Accuracy = 0.978 GLUE Human baseline^B^ = 0.978	*Assessing depression* using Twitter data (from a shared task (CLPsych 2015 (Coppersmith et al., 2015). *N* = 327, test set *n* = 150	Large language models (MentalRoBERTa^4,^ see also^5^) RoBERTA fine tuned on mental health related Reddit data.	F1 = 0.697
Paraphrase (MRPC) *Is sentence B a paraphrase of sentence A?*	Large language model decoding-enhanced BERT with disentangled attention (DeBERTa / Turing NLR v4^2^)	F1 = 0.940 Accuracy = 0.920 GLUE Human baseline^B^ = 0.863/0.808	*Assessing suicide risk* using Reddit data from the SuicideWatch online forum (*Suicide forums only*), and users’ other Reddit posts (*Suicide* + *all forums*) *N* = *621*	*Suicide forums only*: Large language models (Multifeature Fusion Attention Network)^6^ *Suicide* + *all forums*: Large language models with multi-level dual-context language and BERT^7^	*Suicide forums only*: F1 = 0.514 *Suicide* + *all forums:* F1 = 0.457
Similarity (STS-B) *How similar are the two sentences A and B?*	Large language models with efficient denoising pretraining (METRO / Turing NLR v5^3^)	Pearson *r* = 0.937 GLUE Human baseline^B^ = 0.927	*Assessing personality* from social media (Facebook) language *N* = 68,687, test set *n* = 1943	Large language models (word- and message-level attention in combination with past approaches)^9^	Disattenuated Pearson *r* = 0.54–.66
Acceptability (CoLA) *Is a sentence grammatical or ungrammatical?*	Large language models for large-scale knowledge enhanced pre-training (EARNIE^1^)	Mathew’s Correlation = 0.738 GLUE Human baseline^B^ = 0.664	*Assessing well-being (harmony in life)* from probed language *N* = 608	Large language models (BERT)^8^	Pearson *r* = 0.85 Dissattenuated Pearson *r* = 1.00

Notes. SST-2 = The Stanford Sentiment Treebank; MRPC = The Microsoft Research Paraphrase Corpus; STS-*B* = The Semantic Textual Similarity Benchmark; CoLA = The Corpus of Linguistic Acceptability.

A Top performing systems are selected from the GLUE leaderboard (https://gluebenchmark.com/leaderboard), where the system needs to be in top 50 overall and be described with a URL and accompanied with a manuscript describing the system.

B GLUE Human baseline = “a conservative estimate of human performance”, where the participants/annotators were non-experts recruited through crowdsourcing (Nangia and Bowman, 2019).

1 ERNIE (https://github.com/PaddlePaddle/ERNIE (Sun et al., 2021)

2 DeBERTa (https://github.com/microsoft/DeBERTa (He et al., 2021)

3 METRO / Turing NLR v5 (https://arxiv.org/abs/2204.06644, (Bajaj et al., 2022)

4 MentalRoBERTa (Ji et al., 2021)

5 (Matero et al., 2021) shows that RoBERTA performs more accurately than non-transformers

6 (Li et al., 2021)

7 (Matero et al., 2019)

8 (Kjell et al., 2022)

9 (Lynn et al., 2020).

## Footnotes

1 Still, Latent Semantic Analysis (Deerwester et al., 1990), a bag-of-word approach representing words with numeric representations, was developed by psychology researchers in the 1990s.

2 Participants were recruited online from Prolific, an online platform for recruiting participants that are being paid to take part in the research. Age: M = 41.3, SD = 14.6, range = 18 – 88 years; Gender: females = 58, males = 42; Nationality = U.K.

3 The Self-Information Diversity Index is a measure of diversity or entropy in a set of numeric values or probabilistic events (i.e., possible responses on a scale or by words). It is 2^entropy^, where entropy is computed as the Shannon entropy, mathematically defined as: H=−∑xp(x)log(p(x)), where p is its probability mass function, and x is a set of numeric values or probabilistic events.

4 Exactly how to partition words into discrete senses is hotly debated in linguistics, but fundamentally, the context of a word is essential to its meaning. For more on the debate see (Navigli, 2009). Modern AI has cleverly side-stepped the sense-partitioning debate by representing meaning in a latent semantic space – similar to factors derived from factor analysis as we discuss in the next section.

5 A recurrent neural network (RNN) might be able to get the same performance if it could parallelize its training routine in the same way transformers do.

6 In these tasks, the test answers are not available to the public, so researchers submit their models’ answers for testing, and the results are then presented publicly on leaderboards (https://gluebenchmark.com/leaderboard)

7 The NLP analyses were done in R using the text-package. First, individuals’ text responses to how they were feeling were transformed into word embeddings using the large language models, roberta-large. Then, the affective valence of each response was estimated using an AI valence estimator trained on an open affect dataset (Preoţiuc-Pietro et al., 2016), with our estimator having a cross-validated accuracy with raters of Pearson r =.74. Lastly, the responses were divided into those that were positive and negative and then summarized using the large language model t5-large. See the open code for details.

8 From season 1, episode 12.
